# Supplementary material for: Regenerative biologics modulating inflammation and promoting tenogenesis in equine superficial digital flexor tendonitis: from molecular pathways to clinical translation
Source: Ir Vet J. 2025 Sep 17;78:21. doi: 10.1186/s13620-025-00309-z (PMC12442297; doi:10.1186/s13620-025-00309-z)
Supplement: Supplementary file 1 — Supplementary Material 1. [file 13620_2025_309_MOESM1_ESM.docx]

**Regenerative biologics modulating inflammation and promoting tenogenesis in equine superficial digital flexor tendonitis: from molecular pathways to clinical translation**

**Mahmoud Najeb, Alaa Samy, Awad Rizk, Esam Mosbah, Gamal Karrouf***

Department of Surgery, Anesthesiology, and Radiology, Faculty of Veterinary Medicine, Mansoura University, 35516 Mansoura, Egypt.

*Correspondence: Gamal Karrouf; Department of Surgery, Anesthesiology, and Radiology, Faculty of Veterinary Medicine, Mansoura University, 35516 Mansoura, Egypt. Tel: +201009016696, E-mail address: [drgamalkarrouf1966@gmail.com](mailto:drgamalkarrouf1966@gmail.com), gamalkarrouf@mans.edu.eg

**Abstract**

Superficial digital flexor (SDF) tendonitis is a major cause of lameness and early retirement in equine athletes. Research has shifted the understanding of tendonitis from being only a degenerative condition to recognizing inflammation as a central and dynamic factor in both its development and repair. While regenerative therapies such as platelet-rich plasma and mesenchymal stem cells have demonstrated promising benefits, their clinical efficacy remains inconsistent, and no single gold-standard protocol has yet emerged. This review highlights the inflammatory concept of equine tendonitis, with a focus on cellular and molecular mechanisms behind tenogenesis. In addition, we explore the emerging evidence supporting regenerative biologic interventions in modulating inflammation and promoting tenogenesis. With ongoing advances in the understanding of tendon pathobiology, this review highlights inflammation as a central determinant of tendon healing outcomes and outlines promising therapeutic avenues, such as autologous conditioned serum, injectable platelet-rich fibrin, and mesenchymal stem cells-derived exosomes, for SDF tendonitis in equine.

**Keywords:** Superficial digital flexor tendon, tendon healing, inflammation, biologic therapies, equine.

***Introduction***

Tendon injuries, particularly those affecting the superficial digital flexor tendon (SDFT), are a prevalent and debilitating issue in performance horses, accounting for up to 72% of lost training days and a significant proportion of early retirements **(1-3)**. Equine superficial digital flexor (SDF) tendonitis poses both clinical and economic burdens, with incidence rates reported to range from 11-46% of all limb-related injuries **(4, 5)**. The inherent biomechanical vulnerability of SDFT, combined with its limited vascularity, high mechanical demands, and poor intrinsic cellular healing capacity, results in a healing response characterized by disorganized fibrosis rather than true regeneration **(6)**. Once severely damaged, tendons exhibit a limited ability to restore their native elasticity and biomechanical strength, resulting in a reinjury rate of up to 80% following conservative management **(7-9)**.

A wide range of therapeutic interventions has been proposed, and continue to emerge for treatment of SDF tendonitis in equine patients. This ongoing development of treatment options reflects the complexity of pathophysiology and the persistent challenges in determining the evidence for treatment efficacy **(10)**.

Recent advances in regenerative medicine have introduced a wide range of biologic therapies aimed at promoting tendon repair, including mesenchymal stem cells and platelet-derived products **(11, 12)**. However, clinical outcomes remain variable, and true regenerative healing is often not achieved **(10)**. For instance, among platelet-derived products, platelet-rich plasma (PRP) has received significant attention and has shown beneficial effects in experimental models **(13, 14)**. However, its efficacy in equine tendon healing remains controversial. Two systematic reviews encompassing over thirty studies reported improvements in lameness, tissue healing, and return-to-performance rates **(15, 16)**. In contrast, a recent meta-analysis of fifteen studies found no definitive evidence that PRP significantly enhances tendon healing outcomes in horses **(17)**.

One of the emerging explanations for this inconsistency is the failure to adequately control the inflammatory cascade during tendon healing **(18-20)**. Persistent or unresolved inflammation not only delays the transition to the reparative phases but may also compromise the therapeutic efficacy of regenerative interventions **(21, 22)**. Inflammation initiates and coordinates the healing process, but its timely regulation is essential to prevent chronic damage and promote optimal tendon recovery. Inflammatory dysregulation can alter the local microenvironment, leading to cellular dysfunction, matrix degradation, and ultimately, fibrotic healing rather than functional tissue regeneration **(20)**. This aligns with existing concepts of failed healing, where we believe there is a deficiency in properly switching off the inflammatory process.

Chronic inflammation, marked by cytokines like interleukin-1β (IL-1β) and tumor necrosis factor-alpha (TNF-α), sustains the production of disorganized collagen type III, impeding the transition to mature, organized collagen type I and leading to poor tissue structure and function **(15)**. Inflammatory environments increase matrix metalloproteinases (MMPs) activity, accelerating collagen degradation and further disrupting matrix integrity **(23)**. The balance between pro-inflammatory (M1) and anti-inflammatory (M2) macrophages is also crucial. M1 macrophages promote inflammation and matrix breakdown, while M2 macrophages support tissue repair and collagen maturation **(24)**.

Regenerative biologics possess potent immunomodulatory and regenerative properties, enabling them to downregulate pro-fibrotic cytokines, modulate key molecular pathways, and promote macrophage polarization toward the reparative M2 phenotype, thereby enhancing the healing process **(25)**. A comprehensive understanding of the inflammatory cascade enables the strategic selection, timing, and potential combination of these therapies to maximize their therapeutic potential. Such an approach is critical for the precise modulation of inflammation, ultimately improving treatment outcomes and promoting true tendon regeneration **(26)**.

This review aims to explore the biological interplay between inflammation and tenogenesis, and explore the role of regenerative biologic therapies to modulate inflammation and promote tenogenesis, particularly in equine SDF tendonitis. Among the regenerative biologics addressed in this review are platelet-derived products (including PRP and platelet-rich fibrin [PRF]), autologous conditioned serum (ACS), autologous protein solution (APS), autologous conditioned plasma (ACP), mesenchymal stem cells (MSCs), stromal vascular fraction (SVF), bone marrow aspirate concentrates (BMAC), and MSCs-derived exosomes. It is important to state, however, that this review does not follow a structured, systematic methodology such as PRISMA. Instead, it was conducted as a narrative synthesis, grounded in a targeted selection and descriptive analysis of the commonly used and clinically relevant therapeutic biologics.

**Pathophysiology of Equine Tendonitis**

The SDFT is highly susceptible to tendonitis due to its role as an energy-storing tendon and exposure to mechanical overload that exceeds the structural tolerance of the tissue **(27)**. This overload may result from sudden excessive stretching or, more commonly, from the cumulative effects of repetitive strain **(28)**. Microdamage accumulates gradually within the collagen matrix, and most lesions develop subclinically before clinical signs appear **(29)**. Clinically, acute SDFT injuries present with variable lameness, and in severe cases, fetlock hyperextension may be observed due to loss of tendon integrity **(18)**. Although clinical signs may resolve, inflammation persists at the molecular level, indicating ongoing cellular pathology **(30)**.

Both ageing and repeated mechanical loading disrupt tendon structure by inducing collagen disorganization and matrix protein imbalance **(31, 32)**. Studies have revealed age-related accumulation of degradation fragments and altered glycosaminoglycan profiles, contributing to reduced mechanical resilience and higher reinjury risk **(33, 34)**.

Tendon repair begins with type III collagen deposition, forming a mechanically weaker matrix than native type I collagen. During remodeling, partial replacement by type I collagen occurs, aided by fibroblast-mediated contraction and alignment **(35)**. However, the repaired tissue seldom restores full strength or organization, making controlled, programmed exercise essential to guide fiber alignment and enhance functional recovery **(10)**.

**Pro-inflammatory Cytokines and Molecular Pathways Regulating Tendon Healing & Macrophage Polarization**

During the acute phase of tendon injury, the release of damage-associated molecular patterns (DAMPs), alarmins, from necrotic tendon cells initiates a robust type 1 immune response, primarily mediated by resident tenocytes and infiltrating immune cells **(35, 36)**. These DAMPs activate pattern recognition receptors (PRRs) such as Toll-like receptors (TLRs) on both cell types, triggering rapid secretion of pro-inflammatory cytokines, including IL-1β and TNF-α, which are significantly upregulated in animal models during acute phases, pointing to their role in initiating the early inflammatory cascade **(23, 37-39)**. These cytokines not only upregulate matrix-degrading enzymes such as MMPs, but also strongly activate intracellular signaling cascades, most notably the nuclear factor kappa-light-chain-enhancer of activated B cells (NF-κB) pathway **(40-43)**. This type 1 immune response helps in initiating tissue repair through immune cell recruitment, matrix clearance, and activation of reparative processes. However, if not properly regulated, it sustains a state of chronic inflammation that disrupts extracellular matrix (ECM) synthesis, promotes tenocyte apoptosis or dysfunction, and impairs functional recovery **(21, 22)**. In order to prevent the excessive pro-inflammatory response of the type I immune response, the body activates the type II immune response for anti-inflammation. This anti-inflammatory phase is orchestrated by cytokines such as IL-4 and IL-33, which are released from damaged or activated stromal cells and promote the polarization of macrophages toward the M2 phenotype **(44)**. Additionally, regulatory T cells (Tregs) secrete IL-10, a key cytokine that suppresses type 1-mediated inflammation and facilitates resolution **(41)**. This shift is crucial to terminate the inflammatory phase, restore immune balance, and create a regenerative environment conducive to matrix repair **(Figures 1 & 2)** **(45)**.

In addition to the canonical cytokine-mediated inflammation, several intracellular signaling pathways play pivotal roles in orchestrating the inflammatory response during tendinopathy. Among these, the NF-κB pathway is the most extensively studied and serves as a powerful pro-inflammatory signaling pathway. Upon activation by cytokines such as IL-1β and TNF-α, NF-κB translocates to the nucleus and promotes transcription of pro-inflammatory genes, including IL-6 and cyclooxygenase-2 (COX-2), thereby sustaining the inflammatory milieu and contributing to matrix degradation and fibrosis **(43, 46)**. Under hypoxic conditions, NF-κB activation stabilizes hypoxia inducible factor-1α (HIF-1α) and promotes reactive oxygen species (ROS) production, forming a self-amplifying inflammatory loop that disrupts matrix homeostasis and drives chronic tendon degeneration **(41)**. Recent in vivo evidence demonstrated that canonical NF-κB activation persists beyond the inflammatory phase and promotes myofibroblast survival during the remodeling stage of tendon healing, thereby contributing to fibrotic matrix deposition rather than true regeneration **(30)**.

The nucleotide-binding domain, leucine-rich–containing family, pyrin domain–containing-3 (NLRP3) inflammasome represents another critical inflammatory mechanism. Upon stimulation by danger signals such as high mobility group box 1 (HMGB1), mitochondrial dysfunction, or ionic flux (K⁺ efflux, Ca²⁺ influx), NLRP3 assembles with apoptosis-associated speck-like protein containing a caspase recruitment domain (ASC) and caspase-1 to promote the maturation of IL-1β and IL-18 **(47, 48)**. This pathway contributes to excessive ECM remodeling and inflammatory cell recruitment, and its overactivation has been associated with poor tendon healing and fatty infiltration of tenocytes **(49)**.

The p38 mitogen-activated protein kinase (MAPK) pathway is activated by mechanical stress and oxidative signals, and modulates cellular responses by promoting transcription of TNF-α, IL-6, and IL-8 **(50)**. Persistent activation contributes to heterotopic ossification and chronic inflammation, while pharmacologic inhibition has shown promise in reducing these adverse outcomes in preclinical models **(51, 52)**.

The Janus kinase/signal transducer and activator of transcription 3 (JAK/STAT3) signaling pathway also plays a dual role in tendon inflammation. While it may contribute to fibrosis and senescence when persistently activated **(53)**, it is also essential for mediating the anti-inflammatory effects of IL-10 **(54, 55)**. Activation of the interleukin-10 receptor/Janus kinase/signal transducer and activator of transcription 3 (IL-10R/JAK/STAT3) axis downregulates NF-κB activity and promotes M2 macrophage polarization, thereby limiting excessive ECM deposition and supporting regenerative remodeling **(56)**.

A key player in the tendon healing process is the macrophage, due to its ability to dynamically switch phenotypes in response to environmental cues **(57)**. Initially, pro-inflammatory M1 macrophages dominate the injury site to clear debris and promote inflammation, but a timely transition to the anti-inflammatory M2 phenotype is critical for resolving inflammation and promoting tissue repair. However, disruption of this transition can lead to chronic inflammation **(57-59)**. Importantly, M2 macrophages secrete anti-inflammatory mediators such as IL-10 and interleukin-1 receptor antagonist (IL-1Ra), which are key to suppressing the early inflammatory response, preventing further tissue degradation, and initiating a reparative immune response **(24)**. This reparative response attracts growth-promoting factors and may continue throughout the proliferative and remodeling stages, providing a supportive microenvironment for tissue repair **(22)**.

**Cross-communication in Tendon Microenvironment**

The tendon microenvironment is characterized by cross-talk between immune cells and tendon-resident cells, including tenocytes and progenitor/stem cells, through exchange of exosomes and soluble mediators **(60)**. This intricate communication plays a central role in controlling the overall healing process **(61, 62)**. In an autologous indirect tenocytes co-culture with peripheral blood mononuclear cells (PBMCs), the presence of PBMCs led to a marked upregulation of proinflammatory cytokine gene expression, including IL-1β, TNF-α, and IL-6. This suggests that the crosstalk between tenocytes and immune cells occurs via secreted factors **(63)**. Experimental data have shown that tenogenic markers such as scleraxis, along with cell proliferation capacity, were significantly reduced when MSCs were cultured in the presence of immune cells **(64)**.

Macrophages, among the key immune regulators, particularly through their dynamic polarization, represent a highly coordinated and adaptable process tightly regulated by a broader array of factors from the local microenvironment, including tissue-specific molecular cues, differentiation signals, and interactions with neighboring cell types’ collective cues **(24, 59, 65, 66)**. In particular, signals from tendon progenitor cells, especially CD146⁺ cells, actively contribute to the resolution of inflammation by producing anti-inflammatory molecules such as IL-10 and tissue inhibitor of metalloproteinases-3 (TIMP-3), as well as extracellular vesicles like exosomes, which carry regulatory microRNAs and proteins that influence macrophage polarization **(67, 68)**. However, persistent pro-inflammatory signals, like IL-1 or DAMPs, can disrupt this transition. These signals not only keep macrophages in the M1 state but also impair the function of surrounding progenitor cells, limiting their capacity to produce regenerative signals **(Figure 3)** **(60, 69)**. Moreover, the signaling pathways associated with the activation of the M1 or M2 phenotypes include the pro-inflammatory pathways involving interferon gamma (IFN-γ) and NF-κB, and inflammation-resolving pathways mediated by glucocorticoid receptor activation **(70)**.

Additional evidence has identified diverse immune components within the tendon microenvironment, including tenophages, mast cells, T and B lymphocytes, and natural killer cells, all of which contribute to the regulation of inflammation during tendon healing **(66, 71, 72)**.

Tendon cells, including tenocytes and tendon stem/progenitor cells, are highly mechanosensitive; they transduce external loading into intracellular signals that regulate cell behavior, matrix synthesis, and immune responses **(73)**. When exposed to physiological loading, such as that induced by controlled exercise, they enhance the synthesis of collagen and activate cross-linking enzymes, thereby improving tendon tensile strength **(74)**. In contrast, mechanical unloading, which may result from immobilization or inactivity, suppresses the expression of key extracellular matrix components, ultimately compromising tendon structural integrity **(75)**. These load-dependent cellular behaviors are orchestrated through mechanotransduction pathways involving the actin cytoskeleton and transcriptional regulators, which translate matrix stiffness into gene expression programs that promote either tissue stiffening or elasticity **(76)**. As such, maintaining a balanced mechanical environment is essential, not only for directing tenogenic responses but also for preventing pathological outcomes like fibrosis **(73)**.

The tendon microenvironment operates as a highly integrated regulatory network, where immune, stromal, and mechanical signals interact through overlapping pathways **(26, 77)**. This complexity underlies the limited efficacy of single-target therapies and supports the development of multimodal strategies that address the diverse regulatory axes involved in tendon healing **(78)**.

**Conventional Anti-inflammatory Therapies: Mechanisms and Limitations**

In the management of SDFT overstrain injuries, where pain is not a dominant chronic feature, therapeutic strategies are aligned with the distinct phases of tendon healing **(Table 1)**, aiming primarily to restore function rather than merely relieve pain **(10)**. During the acute inflammatory phase, inflammation should be modulated, not entirely suppressed **(79)**. Early cytokine activity and proteolytic enzymes are essential for debris clearance and initiate repair signaling, but prolonged or excessive inflammatory signaling can disrupt healthy matrix and promote fibrosis **(80)**. Thus, a balanced approach that permits initial inflammatory activation followed by timely resolution is optimal for favorable tendon healing **(10)**.

Conventional anti-inflammatory treatments like Non-steroidal anti-inflammatory drugs (NSAIDs), cold therapy, compression, and topical Dimethyl sulfoxide (DMSO) are widely used to treat intratendinous inflammation and relieve pain, but these primarily suppress inflammation rather than promote its natural resolution **(81)**. The use of corticosteroids remains controversial due to their potential to impair tenocyte function, and if used, they should be limited to the early inflammatory phase and applied peritendinously rather than intratendinously **(10)**.

**Modulating Inflammation for Regenerative Outcomes**

Emerging evidence suggests that transitioning from merely suppressing inflammation to actively modulating and resolving it represents a more physiological and regenerative approach to tendonitis treatment **(20)**. Recent findings highlight that specific elements of the inflammatory cascade are essential not only for initiating repair but also crucial for proper resolution of tissue injury **(82)**. In this context, ACS has shown promise due to its enrichment with IL-1Ra, which competitively inhibits IL-1 binding to its receptors **(83, 84)**. This targeted blockade disrupts IL-1–mediated recruitment of M1 macrophages and reduces the expression of matrix-degrading enzymes such as MMPs **(85).**

Although ACS does not directly induce macrophage polarization toward the reparative M2 phenotype, its capacity to attenuate the pro-inflammatory environment facilitates a shift toward tissue regeneration **(61)**. This permissive environment allows immune and progenitor cells to regain regulatory function, leading to the secretion of exosomes and anti-inflammatory mediators such as IL-10 and transforming growth factor beta (TGF-β), which further promote M2 polarization and resolution of inflammation **(62).**

ACS has shown promise in enhancing tendon healing, particularly in experimental models and some clinical studies in equine patients **(84)**. In animal models, ACS treatment led to improved histological healing, increased type I collagen expression, and accelerated recovery of tendon structure, although improvements in ultimate tendon strength were not consistently observed within the study periods **(86-88)**. Notably, a single intralesional injection of ACS in horses diagnosed with SDF tendonitis was shown to reduce lameness and swelling, improve ultrasonographic and histological outcomes, and enhance collagen type I expression **(89)**. Similarly, APS, which shares a comparable biological profile with ACS, demonstrated protective effects against IL-1-mediated matrix degradation **(85)**. Moreover, APS treatment significantly reduced collagen type III expression in an equine SDF tendonitis model, further supporting its potential role in enhancing tendon matrix composition and mechanical integrity **(90)**. On the other hand, an in vitro study on equine tenocytes demonstrated that ACS did not fully reverse the detrimental effects induced by combined IL-1β and TNF-α stimulation **(40)**.

While experimental and clinical data suggest that ACS can enhance histological repair, increase type I collagen expression, and alleviate clinical signs in equine tendon injuries **(84)**, its clinical utility remains a topic of debate among equine practitioners **(91)**. The biological effect of ACS relies primarily on IL-1Ra–mediated blockade of the IL-1 pathway, which limits its capacity to modulate other key inflammatory cascades such as NF-κB **(40)**. This narrow target profile may contribute to persistent inflammation or the inconsistent improvements in ultimate tensile strength reported in some studies **(86-88)**. Furthermore, histological improvements often precede measurable recovery of mechanical properties, and the absence of cellular scaffolds may reduce long-term regenerative potential compared with treatments like I-PRF **(90)**. Finally, the short follow-up periods in most equine studies restrict the ability to draw strong conclusions on recurrence rates or long-term performance outcomes.

Other biological therapies, such as PRF, have been shown to enhance antioxidant defenses in tenocytes and to promote macrophage polarization toward the anti-inflammatory M2 phenotype, as demonstrated in controlled macrophage cultures **(92-94)**. However, direct evidence confirming these immunomodulatory effects within the tendon microenvironment, particularly on resident tendon macrophages, remains limited.

Similarly, exosomes derived from MSCs have been reported to promote macrophage polarization toward a reparative M2 phenotype and downregulate pro-inflammatory and apoptotic markers **(95-99)**. In human PBMCs, MSCs-derived exosomes suppress key pro-inflammatory cytokines (IL-1β, TNF-α) while enhancing anti-inflammatory mediators such as IL-10 and TGF-β **(100)**. Priming MSCs with IL-1β further augments these effects by enriching exosomes with microRNA (miR) such as miR-147b, which suppresses IL-1β/TNF-α expression and inhibits NF-κB signaling **(101, 102)**. Complementary explant and co-culture studies support translational relevance, showing that MSCs-derived exosomes reduce IL-6 and MMP-3, and exert anti-inflammatory effects in tenocyte–macrophage co-cultures **(103, 104)**. However, an in vitro study reported limited anti-inflammatory activity when equine tenocytes were directly stimulated with IL-1β and TNF-α **(40)**.

While MSCs-derived exosomes have demonstrated promising results in preclinical tendonitis models, the absence of clinical studies in naturally occurring equine tendon injuries limits the strength of current recommendations **(105)**. Their occasional limited efficacy in highly inflammatory environments may be related to the inability to modulate all key inflammatory pathways, particularly under strong IL-1β and TNF-α stimulation **(40)**. Considering these constraints, exosomes derived from tenogenic-primed MSCs or primed with IL-1β may represent a promising alternative or adjunct, offering greater functional specificity and a more targeted pro-regenerative effect in tendon repair **(106)**.

**Promoting Tenogenesis**

Biological therapies currently represent the most commonly employed strategy for promoting tenogenesis in equine SDF tendonitis. These approaches rely on products derived from blood or tissues to elicit a regenerative healing response **(15)**. Although the definitive goal of tissue regeneration remains unachieved with current strategies **(10)**, numerous experimental and clinical studies have demonstrated improved healing quality and favorable clinical outcomes following the application of these biological therapies **(96, 107-116)**.

Most biologics used fall into two major categories, growth factor-based products and cellular therapies. While individual growth factors such as IGF-1 have been explored, platelet-derived products (PDPs) and various preparations of ACS are more commonly applied due to their content of a broader mixture of bioactive molecules **(13, 84, 85, 90)**.

Among PDPs, PRP has received significant attention and shown beneficial effects in experimental models **(13, 14)**. However, its efficacy in equine tendon healing remains controversial both experimentally and in clinical trials **(10)**. Although several individual studies have reported favorable histological or clinical improvements following PRP administration in equine tenodesmic lesions **(15, 16)**, the recent meta-analysis pooling fifteen trials found no definitive evidence of benefit over controls **(17)**.

Autologous conditioned plasma (ACP), a leukocyte-reduced platelet concentrate prepared stall-side by a simplified single-spin centrifugation, offers a moderate platelet enrichment (~1.2–2.5× baseline) with markedly lower white blood cell content compared to many PRP protocols **(117, 118)**. In equine practice, a small case series reported clinical improvement following intralesional ACP injections for severe tendinitis; however, the absence of controls and standardized outcome measures limited the strength of these findings **(119)**. In a surgically induced SDF Tendonitis model, two ACP injections did not yield significant improvements in ultrasonographic, histological, or biomechanical outcomes over saline controls at 24 weeks, despite modest biochemical changes **(120)**.

Variability in PRP preparation protocols, including platelet concentration, leukocyte content, and activation methods, significantly influences the biological activity and subsequent healing outcomes **(121)**. For instance, leukocyte-rich PRP may provoke a heightened inflammatory response, potentially delaying regeneration, whereas leukocyte-poor formulations might favor anti-inflammatory effects but lack sufficient growth factor release **(122)**. Methodological limitations in existing studies, including small sample sizes, lack of proper controls, and inconsistent outcome measures, limit the reliability of positive findings and underscore the need for well-designed, standardized clinical trials **(12)**. Compared to other biologics like MSCs may fall short in providing consistent regenerative benefits **(123)**.

PRF, an advanced platelet-derived product, has gained attention as a favorable substitute to PRP. It can be simply prepared stall-side from autologous blood without the need for anticoagulants or sophisticated laboratory tools. Compared to PRP, PRF provides a greater amount of cytokines and growth factors and ensures a more prolonged release, lasting up to fourteen days versus nearly four days in PRP **(124, 125)**. Moreover, its fibrin-rich network facilitates cellular migration and serves as a supportive scaffold, while being free of chemical additives that may hinder its therapeutic efficacy **(126)**. The liquid form of platelet-rich fibrin, also known as injectable PRF (I-PRF), permits intratendinous administration, unlike the conventional clotted PRF. Our previous study utilized I-PRF in the treatment of naturally occurring superficial digital flexor tendonitis in donkeys and demonstrated significant improvements in clinical outcomes compared to saline-treated controls **(127)**.

A key element for effective tendon repair and regeneration is the availability of a suitable cellular source. Such cells are essential to leverage their proliferative capacity, contribution to intercellular signaling, secretion of bioactive molecules, and support of ECM synthesis **(128)**. Stem cells, in particular, have attracted considerable interest owing to their intrinsic multipotency **(129)**.

Among the different stem cell types, MSCs are an attractive cell source due to their high proliferative ability and capacity to differentiate into multiple cell types. MSCs are commonly derived autologously, especially from bone marrow or adipose tissue **(114, 123)**. Allogeneic MSC products have also been safely employed in clinical settings **(112, 130)**, though concerns persist regarding potential immune responses to non-autologous sources **(131, 132)**. In addition, stem cells have also been isolated from tendon tissue itself across several species, including horses **(133)**. These tendon-derived stem cells have shown promising regenerative potential and may offer advantages in terms of tenogenic differentiation when applied to tendon repair **(134)**.

Despite their regenerative potential, the clinical application of MSCs in tendon therapy faces several limitations. Maintaining MSCs' viability after administration is difficult, particularly in the harsh inflammatory tendon microenvironment, with studies showing less than 5% survival of MSCs within 10 days post-injection **(105, 135)**. There are also concerns about immune rejection and potential tumorigenicity, particularly with prolonged culture or repeated administration **(131, 132)**. Multiple clinical studies show that intralesional MSCs can improve return-to-racing rates and reduce reinjury **(113, 114, 116)**. However, a recent meta-analysis found their effectiveness to be inconclusive, largely due to heterogeneity in cell sources, preparation and priming methods, timing of administration, and outcome measures, as well as generally small sample sizes, short follow-up periods, and high risk of bias in most studies **(12)**.

The therapeutic effects of MSCs appear to be primarily mediated through paracrine mechanisms rather than direct cellular replacement **(136, 137)**. In particular, increasing evidence supports the role of MSCs-derived extracellular vesicles (EVs) as key modulators of the tendon healing microenvironment **(105)**. EVs, which include microvesicles and exosomes, are membrane-bound, nanoscale elements that are actively released by almost every type of cell **(138)**. Their bioactive cargo, including microRNA, proteins, lipids, and cytokines, reflects the parent cell's functional state **(Figure 4)** **(137)**. In this way, EVs enable distant cells to interact and modulate each other's function by delivering bioactive signals **(111)**. MSCs-derived exosomes represent a cell-free, immunocompatible alternative to traditional MSC therapies **(138)**. Exosomes are more stable and reservable than cells, have no chance of aneuploidy, and have a lower risk of immune rejection after in vivo allogeneic administration **(6)**.

Various experimental model studies have demonstrated that MSCs-derived exosomes enhance tendon repair **(99)**. These exosomes regulate the balance between ECM synthesis and degradation and promote tendon regeneration by enhancing the proliferation and migration of endogenous tendon stem/progenitor cells, increasing tendon marker expression, collagen I deposition, and improving biomechanical strength **(139, 140)**. Their cargo activates pro-survival signaling pathways, thereby reducing apoptosis and fostering a regenerative microenvironment **(96)**. In a rat model of Achilles tendinopathy, exosomes derived from MSCs enhanced tendon healing by increasing type I collagen expression and improving biomechanical properties. These effects were partly linked to the enrichment of miR-29a, miR-21-5p, and miR-148a-3p, which are known to regulate collagen remodeling and promote tissue regeneration **(97)**. Engineering MSCs-derived exosomes to overexpress miR-29a further amplified these effects **(141)**.

Adipose-derived SVF comprises a heterogeneous mix of cells, stem/progenitor cells, pericytes, and endothelial cells, obtained directly from adipose tissue without cell culture expansion, offering a rapid, cost-effective, and minimally manipulative alternative to culture-expanded MSCs **(142)**. In vitro studies in equine models have shown that SVF exhibits elevated expression of key growth factors such as insulin-like growth factor 1 (IGF-1) and TGF-β, and its conditioned medium promotes tenocyte chemotaxis and ECM-related gene expression, positioning SVF as a potent trophic mediator in tendon healing **(143)**. Experimental in vivo studies, including collagenase- and surgically-induced SDFT lesions in horses, suggest that SVF administration can enhance collagen fiber organization and promote neovascularization **(144)**. A small clinical case report involving three Thoroughbreds with naturally occurring SDF tendinitis found that intralesional autologous SVF injections correlated with marked clinical improvement and a shortened rehabilitation period **(145)**. However, SVF exists in distinct forms (enzymatically vs. mechanically isolated), each with differing cell compositions and bioactivity **(144)**. This heterogeneity, compounded by batch-to-batch variability influenced by donor tissue characteristics and processing protocols, poses a major challenge for standardization.

BMAC, represent a culture-free cellular product containing a mixture of mesenchymal stem/progenitor cells, hematopoietic cells, endothelial progenitors, leukocytes, and platelets, along with an array of bioactive cytokines and growth factors **(146)**. BMAC can be prepared stall-side through point-of-care centrifugation of autologous bone marrow aspirates, offering a rapid and minimally manipulative alternative to culture-expanded MSCs **(147)**. In vitro, BMMNCs exhibit anti-inflammatory, trophic effects, and enhancement of ECM synthesis **(148)**. Clinically, multiple equine case series and retrospective analyses have reported improved ultrasonographic lesion resolution, reduced reinjury rates, and higher return-to-performance in horses treated with intralesional BMAC compared with conventional therapies or PRP **(149, 150)**.

***Combination therapy***

Combining MSCs with PRP has demonstrated synergistic potential in tendon healing. They improve histological architecture, upregulate tenogenic markers, and enhance biomechanical properties in both preclinical rat models and clinical studies in humans **(151-156)**. In horses with SDF tendonitis, co-administration of MSCs and PRP led to superior healing outcomes compared to either therapy alone **(109, 157-159)**, with meta-analyses suggesting reduced reinjury rates, although evidence regarding return to performance remains inconsistent **(12)**.

In donkeys, combining ACS with I-PRF improved clinical and ultrasonographic parameters over I-PRF alone in cases of naturally occurring SDFT lesions **(127)**, further supporting the promise of multi-modal biologic strategies in equine tendon repair.

**Practical Considerations Guiding the Use of Regenerative Biologics in SDF tendonitis**

Given the complex nature of tendon healing, selecting the most appropriate regenerative therapy requires more than an understanding of biological mechanisms. It also demands case-specific clinical judgment. Treatment decisions should consider the stage of healing, the lesion's location (e.g., extrathecal vs. intrathecal), and severity **(18)**. The intralesional application of regenerative biologics should be guided by ultrasonography to ensure accurate delivery into the lesion core, minimizing leakage and maximizing product retention at the target site. These therapies are most effective when administered into well-defined hypoechoic core lesions surrounded by intact tendon tissue (e.g., core lesions) or contained by paratenon (e.g., marginal lesions) **(10)**. Intratendinous injections are not recommended once the lesion is filled with mature fibrous tissue. Although the optimal timing for injection has not been definitively established, it is generally intuitive that tenogenic-inductive biologics (e.g., PDPs) exert their best effect when applied after the resolution of the inflammatory phase and during the early proliferative phase, before extensive fibrous matrix deposition. In contrast, immunomodulatory biologics such as ACS or selected exosomes may be more appropriate during the early inflammatory phase to regulate the initial immune response **(160)**. All injections should be performed aseptically in the weight-bearing limb under sedation and local analgesia of the affected area, including the skin. While finer needles reduce mechanical trauma to the tendon, using gauges smaller than 20G may impair stem cell viability **(161)** and hinder the delivery of viscous products such as liquid PRF **(162)**. Therefore, such small calibers should be avoided in cell-based applications. Key aspects of preparation, administration protocols, timing, and safety considerations for commonly used regenerative biologics are summarized in Table **(2)**.

**Challenges in Clinical Translation of Regenerative Biologics**

The clinical translation of regenerative therapies for equine tendonitis is hindered by intersecting biological, methodological, and technical challenges. Equine tendons, particularly the SDFT with its energy-storing function, operate under extreme mechanical strain that modulates both cellular behavior and immune signaling, and they possess a sparsely vascularized ECM with a distinct immune milieu, resulting in reparative responses that differ fundamentally from those of experimental models or simplified in-vitro systems **(160, 163, 164)**. Even experimental lesions within equine patients themselves (e.g., collagenase or surgically induced injuries) fail to fully replicate the degenerative, multifactorial nature of naturally occurring lesions **(90)**.

Furthermore, the strength of clinical studies is weakened by practical barriers inherent to equine research, including high inter-individual variability in healing response, small sample sizes, owner-driven treatment preferences, and the limited feasibility of conducting randomized controlled trials **(10)**. Addressing these barriers through equine-specific protocols and long-term mechanistic studies is crucial to enhancing the consistency, safety, and clinical efficacy of regenerative therapies.

**Future directions**

Effective tendon regeneration depends on targeted modulation of the inflammatory microenvironment, rather than indiscriminate suppression, together with regenerative stimuli **(165)**. Despite the increasing use of biologic therapies, achieving complete and sustained resolution of tendon inflammation remains challenging **(10)**. Current biologics often focus on single cytokines, yet tendon inflammation is driven by complex and redundant signaling pathways **(166).**

Given the multifactorial nature of tendon inflammation and repair, combination biologic therapies may offer a more comprehensive strategy by providing multi-target, optimized modulation of inflammation while simultaneously delivering regenerative cues. Early studies suggest that such synergistic approaches could enhance therapeutic outcomes beyond what is achievable with single-modality treatments **(12)**. Future research should therefore explore rational combinations of biologics, optimized in both timing and dosing, to maximize their efficacy and clinical relevance.

Although stem cell-based therapies are now well established in equine tendinopathy, key challenges remain, particularly related to poor cell retention and limited understanding of their mechanisms of action **(6)**. In this context, increasing attention has been directed toward extracellular vesicles, such as exosomes, which mediate many of the paracrine effects of stem cells. However, their composition is highly variable and requires better product characterization, as well as optimization of dose and timing **(167, 168)**. Therefore, specific regulatory standards are needed to ensure safety, efficacy, and responsible clinical use.

Advancing the clinical application of regenerative biologics for equine tendonitis requires stronger evidence supported by well-designed studies. Although randomized controlled clinical trials remain the gold standard for demonstrating therapeutic efficacy, their feasibility in equine medicine is often limited by ethical concerns, owner preferences, and logistical challenges associated with enrolling sufficiently large and unbiased populations **(10)**. As a practical alternative, large-scale prospective cohort studies, with clearly defined inclusion criteria, standardized treatment protocols, and long-term follow-up, can offer a more achievable and ethically sound pathway for generating meaningful clinical evidence.

**Abbreviations**

Autologous conditioned serum (ACS)

Extracellular vesicles (EVs)

Extracellular matrix (ECM)

Interleukin 1β (IL-1C)

Interferon gamma (IFN-γ)

Interleukin 1 receptor antagonist (IL-1Ra)

Injectable platelet-rich fibrin (I-PRF)

Insulin-like growth factor 1 (IGF-1)

Mesenchymal stem cells (MSCs)

Peripheral blood mononuclear cells (PBMCs)

Platelet-rich fibrin (PRF)

Platelet-rich plasma (PRP)

Superficial digital flexor tendon (SDFT)

Superficial digital flexor (SDF)

Stromal vascular fraction (SVF)

Tumour necrosis factor alpha (TNF-α)

Transforming growth factor beta (TGF-β)

**Author contribution**

**Mahmoud Najeb:** Conceptualization, investigation, methodology, and writing.

**Alaa Samy, Awad Rizk, Esam Mosbah, and Gamal Karrouf:** Review and editing of the article.

**Funding**

Open access funding provided by The Science, Technology & Innovation Funding Authority (STDF) in cooperation with The Egyptian Knowledge Bank (EKB).

**Data availability**

All data generated or analyzed are included in this published article.

**Ethics Declarations**

**Ethics statement**

Not applicable in this review.

**Consent for publication**

All authors confirm their consent for publication.

**Conflict of interest**

No conflicts of interest have been declared.

**Acknowledgements**

Not applicable. The authors declare that they have not used AI-generated work in this manuscript.

**References**

1. Olivier A, Nurton J, Guthrie A. An epizoological study of wastage in thoroughbred racehorses in Gauteng, South Africa. Journal of the South African Veterinary Association. 1997;68(4):125-9.

2. Lam K, Parkin T, Riggs C, Morgan K. Descriptive analysis of retirement of Thoroughbred racehorses due to tendon injuries at the Hong Kong Jockey Club (1992–2004). Equine veterinary journal. 2007;39(2):143-8.

3. Dabbene I, Bullone M, Pagliara E, Gasparini M, Riccio B, Bertuglia A. Clinical findings and prognosis of interference injuries to the palmar aspect of the forelimbs in Standardbred racehorses: A study on 74 cases. Equine Veterinary Journal. 2018;50(6):759-65.

4. Bertuglia A, Bullone M, Rossotto F, Gasparini M. Epidemiology of musculoskeletal injuries in a population of harness Standardbred racehorses in training. BMC veterinary research. 2014;10:1-10.

5. Alzola R, Easter C, Riggs C, Gardner D, Freeman S. Ultrasonographic‐based predictive factors influencing successful return to racing after superficial digital flexor tendon injuries in flat racehorses: a retrospective cohort study in 469 Thoroughbred racehorses in Hong Kong. Equine veterinary journal. 2018;50(5):602-8.

6. Citro V, Clerici M, Boccaccini AR, Della Porta G, Maffulli N, Forsyth NR. Tendon tissue engineering: An overview of biologics to promote tendon healing and repair. Journal of tissue engineering. 2023;14:20417314231196275.

7. Dyson S. Medical management of superficial digital flexor tendonitis: a comparative study in 219 horses (1992‐2000). Equine veterinary journal. 2004;36(5):415-9.

8. O'meara B, Bladon B, Parkin T, Fraser B, Lischer C. An investigation of the relationship between race performance and superficial digital flexor tendonitis in the Thoroughbred racehorse. Equine veterinary journal. 2010;42(4):322-6.

9. Tamura N, Kodaira K, Yoshihara E, Mae N, Yamazaki Y, Mita H, et al. A retrospective cohort study investigating risk factors for the failure of Thoroughbred racehorses to return to racing after superficial digital flexor tendon injury. The Veterinary Journal. 2018;235:42-6.

10. Smith RK. Treatment of tendinopathies. Equine Veterinary Education. 2024;36(12):659-72.

11. Kovac M, Litvin YA, Aliev RO, Zakirova EY, Rutland CS, Kiyasov AP, et al. Gene therapy using plasmid DNA encoding VEGF164 and FGF2 genes: a novel treatment of naturally occurring tendinitis and desmitis in horses. Frontiers in Pharmacology. 2018;9:978.

12. Willow R, Guzmán KE, Panek CL, Colbath AC. Stem cells and platelet-rich plasma for the treatment of naturally occurring equine tendon and ligament injuries: a systematic review and meta-analysis. Journal of the American Veterinary Medical Association. 2024;262(S1):S50-S60.

13. Chalidis B, Givissis P, Papadopoulos P, Pitsilos C. Molecular and biologic effects of platelet-rich plasma (PRP) in ligament and tendon healing and regeneration: a systematic review. International Journal of Molecular Sciences. 2023;24(3):2744.

14. Roshini ST, Arunprasad A, William BJ, Jeyaraja K, Priyadharshini K. Autologous platelet rich plasma for regeneration of tendon injuries in horses. Indian Journal of Animal Research. 2019;53(10):1354-60.

15. Bonilla-Gutiérrez AF, López C, Carmona JU. Regenerative therapies for the treatment of tenodesmic injuries in horses. Journal of Equine Veterinary Science. 2019;73:139-47.

16. Carmona JU, López C. Efficacy of Platelet-Rich Plasma in the Treatment of Equine Tendon and Ligament Injuries: A Systematic Review of Clinical and Experimental Studies. Veterinary Sciences. 2025;12(4):382.

17. Montano C, Auletta L, Greco A, Costanza D, Coluccia P, Del Prete C, et al. The use of platelet-rich plasma for treatment of tenodesmic lesions in horses: a systematic review and meta-analysis of clinical and experimental data. Animals. 2021;11(3):793.

18. Dakin S. A review of the healing processes in equine superficial digital flexor tendinopathy. Equine Veterinary Education. 2017;29(9):516-20.

19. Mosca MJ, Rashid MS, Snelling SJ, Kirtley S, Carr AJ, Dakin SG. Trends in the theory that inflammation plays a causal role in tendinopathy: a systematic review and quantitative analysis of published reviews. BMJ Open Sport & Exercise Medicine. 2018;4(1).

20. Najafi Z, Rahmanian‐Devin P, Baradaran Rahimi V, Nokhodchi A, Askari VR. Challenges and opportunities of medicines for treating tendon inflammation and fibrosis: A comprehensive and mechanistic review. Fundamental & Clinical Pharmacology. 2024;38(5):802-41.

21. Chisari E, Rehak L, Khan WS, Maffulli N. Tendon healing in presence of chronic low-level inflammation: a systematic review. British Medical Bulletin. 2019;132(1):97-116.

22. Arvind V, Huang AH. Reparative and maladaptive inflammation in tendon healing. Frontiers in Bioengineering Biotechnology. 2021;9:719047.

23. Morita W, Dakin S, Snelling S, Carr A. Cytokines in tendon disease: a systematic review. Bone & joint research. 2017;6(12):656-64.

24. Wang Y, Lu X, Lu J, Hernigou P, Jin F. The role of macrophage polarization in tendon healing and therapeutic strategies: Insights from animal models. Frontiers in Bioengineering and Biotechnology. 2024;12:1366398.

25. Pérez Fraile A, González-Cubero E, Martínez-Flórez S, Olivera ER, Villar-Suárez V. Regenerative Medicine Applied to Musculoskeletal Diseases in Equines: A Systematic Review. Veterinary Sciences. 2023;10(12):666.

26. Koch DW. Cellular and Matrix Dynamics of the Equine Tendon. Veterinary Clinics: Equine Practice. 2025.

27. Bardin AL, Taylor NC, Colborne GR. Response of the Thoroughbred forelimb to perturbations caused by a change in ground surface. Journal of Equine Veterinary Science. 2022;112:103897.

28. O’Brien C, Marr N, Thorpe C. Microdamage in the equine superficial digital flexor tendon. Equine Veterinary Journal. 2021;53(3):417-30.

29. Ullah A, Bokhari S, Aslam S, Masood SM, Noor A, Israr UJJJoA, et al. Clinical and sonographic findings in horses affected with chronic flexor tendinopathies and associated soft tissue injuries of various joints. 2022;32(1).

30. Best KT, Nichols AE, Knapp E, Hammert WC, Ketonis C, Jonason JH, et al. NF-κB activation persists into the remodeling phase of tendon healing and promotes myofibroblast survival. Science signaling. 2020;13(658):eabb7209.

31. León F, Mestre A, Priego L. Morphological adaptations in response to chronic exercise across musculoskeletal tissues: a systematic review. Pensar en Movimiento: Revista de ciencias del ejercicio y la salud. 2023;21(2).

32. Zamboulis DE, Marr N, Lenzi L, Birch HL, Screen HR, Clegg PD, et al. The interfascicular matrix of energy storing tendons houses heterogenous cell populations disproportionately affected by aging. Aging and Disease. 2024;15(1):295.

33. Kelly E, Smith R, Dudhia J, Faragher R. Science-in-brief: The importance of senescence in tendinopathy: New opportunities. Equine Vet J. 2020;52(3):349-51.

34. Kwan KYC, Ng KWK, Rao Y, Zhu C, Qi S, Tuan RS, et al. Effect of aging on tendon biology, biomechanics and implications for treatment approaches. International journal of molecular sciences. 2023;24(20):15183.

35. Chartier C, ElHawary H, Baradaran A, Vorstenbosch J, Xu L, Efanov JI, editors. Tendon: principles of healing and repair. Seminars in plastic surgery; 2021: Thieme Medical Publishers, Inc.

36. Crowe LA, McLean M, Kitson SM, Melchor EG, Patommel K, Cao HM, et al. S100A8 & S100A9: Alarmin mediated inflammation in tendinopathy. Scientific reports. 2019;9(1):1463.

37. Manning CN, Havlioglu N, Knutsen E, Sakiyama‐Elbert SE, Silva MJ, Thomopoulos S, et al. The early inflammatory response after flexor tendon healing: a gene expression and histological analysis. Journal of Orthopaedic Research. 2014;32(5):645-52.

38. Müller SA, Todorov A, Heisterbach PE, Martin I, Majewski M. Tendon healing: an overview of physiology, biology, and pathology of tendon healing and systematic review of state of the art in tendon bioengineering. Knee surgery, sports traumatology, arthroscopy. 2015;23:2097-105.

39. Xiang L, Deng H, Zhou S. Effects of TNF-α on Behaviour and Inflammation in Rats with Rotator Cuff Injury through NGF. Discovery medicine. 2024;36(185):1241-9.

40. Smith EJ, Beaumont RE, McClellan A, Sze C, Palomino Lago E, Hazelgrove L, et al. Tumour necrosis factor alpha, interleukin 1 beta and interferon gamma have detrimental effects on equine tenocytes that cannot be rescued by IL-1RA or mesenchymal stromal cell–derived factors. Cell and Tissue Research. 2023;391(3):523-44.

41. Jiang L, Liu T, Lyu K, Chen Y, Lu J, Wang X, et al. Inflammation-related signaling pathways in tendinopathy. Open Life Sciences. 2023;18(1):20220729.

42. Li H, Li Y, Luo S, Zhang Y, Feng Z, Li S. The roles and mechanisms of the NF-κB signaling pathway in tendon disorders. Frontiers in Veterinary Science. 2024;11:1382239.

43. Abraham AC, Shah SA, Golman M, Song L, Li X, Kurtaliaj I, et al. Targeting the NF-κB signaling pathway in chronic tendon disease. Science translational medicine. 2019;11(481):eaav4319.

44. Arvind V, Crosio G, Howell K, Zhang H, Montero A, Huang AH. Functional tendon regeneration is driven by regulatory T cells and IL-33 signaling. Science Advances. 2025;11(17):eadn5409.

45. Eming SA, Wynn TA, Martin P. Inflammation and metabolism in tissue repair and regeneration. Science. 2017;356(6342):1026-30.

46. Best KT, Lee FK, Knapp E, Awad HA, Loiselle AE. Deletion of NFKB1 enhances canonical NF-κB signaling and increases macrophage and myofibroblast content during tendon healing. Scientific reports. 2019;9(1):10926.

47. Thankam FG, Dilisio MF, Dietz NE, Agrawal DK. TREM-1, HMGB1 and RAGE in the shoulder tendon: dual mechanisms for inflammation based on the coincidence of glenohumeral arthritis. PloS one. 2016;11(10):e0165492.

48. Horng T. Calcium signaling and mitochondrial destabilization in the triggering of the NLRP3 inflammasome. Trends in immunology. 2014;35(6):253-61.

49. He Y, Hara H, Núñez G. Mechanism and regulation of NLRP3 inflammasome activation. Trends in biochemical sciences. 2016;41(12):1012-21.

50. Yang Y, Kim SC, Yu T, Yi Y-S, Rhee MH, Sung G-H, et al. Functional roles of p38 mitogen‐activated protein kinase in macrophage‐mediated inflammatory responses. Mediators of inflammation. 2014;2014(1):352371.

51. Barruet E, Morales BM, Cain CJ, Ton AN, Wentworth KL, Chan TV, et al. NF-κB/MAPK activation underlies ACVR1-mediated inflammation in human heterotopic ossification. JCI insight. 2018;3(22):e122958.

52. Wang Q, Yang Q, Zhang A, Kang Z, Wang Y, Zhang Z. Silencing of SPARC represses heterotopic ossification via inhibition of the MAPK signaling pathway. Bioscience reports. 2019;39(11):BSR20191805.

53. Chen M, Xiao L, Dai G, Lu P, Zhang Y, Li Y, et al. Inhibition of JAK-STAT signaling pathway alleviates age-related phenotypes in tendon stem/progenitor cells. Frontiers in Cell and Developmental Biology. 2021;9:650250.

54. Murray PJ. The primary mechanism of the IL-10-regulated antiinflammatory response is to selectively inhibit transcription. Proceedings of the National Academy of Sciences. 2005;102(24):8686-91.

55. Wang Y, He G, Tang H, Shi Y, Kang X, Lyu J, et al. Aspirin inhibits inflammation and scar formation in the injury tendon healing through regulating JNK/STAT‐3 signalling pathway. Cell Proliferation. 2019;52(4):e12650.

56. Liu Y, Wang L, Li S, Zhang T, Chen C, Hu J, et al. Mechanical stimulation improves rotator cuff tendon-bone healing via activating IL-4/JAK/STAT signaling pathway mediated macrophage M2 polarization. Journal of orthopaedic translation. 2022;37:78-88.

57. Xu H-T, Lee C-W, Li M-Y, Wang Y-F, Yung PS-H, Lee OK-S. The shift in macrophages polarisation after tendon injury: a systematic review. Journal of orthopaedic translation. 2020;21:24-34.

58. Sunwoo JY, Eliasberg CD, Carballo CB, Rodeo SA. The role of the macrophage in tendinopathy and tendon healing. Journal of Orthopaedic Research. 2020;38(8):1666-75.

59. Vinhas A, Almeida AF, Gonçalves AI, Rodrigues MT, Gomes ME. Magnetic stimulation drives macrophage polarization in cell to–cell communication with Il-1β primed tendon cells. International Journal of Molecular Sciences. 2020;21(15):5441.

60. Schulze-Tanzil GG, Cáceres MD, Stange R, Wildemann B, Docheva D. Tendon healing: a concise review on cellular and molecular mechanisms with a particular focus on the Achilles tendon. Bone & joint research. 2022;11(8):561-74.

61. Chisari E, Rehak L, Khan WS, Maffulli N. The role of the immune system in tendon healing: a systematic review. British medical bulletin. 2020;133(1):49-64.

62. Russo V, El Khatib M, Prencipe G, Citeroni MR, Faydaver M, Mauro A, et al. Tendon immune regeneration: insights on the synergetic role of stem and immune cells during tendon regeneration. Cells. 2022;11(3):434.

63. Al-Sadi O, Schulze-Tanzil G, Kohl B, Lohan A, Lemke M, Ertel W, et al. Tenocytes, pro-inflammatory cytokines and leukocytes: a relationship? Muscles, ligaments and tendons journal. 2012;1(3):68.

64. Brandt L, Schubert S, Scheibe P, Brehm W, Franzen J, Gross C, et al. Tenogenic properties of mesenchymal progenitor cells are compromised in an inflammatory environment. International journal of molecular sciences. 2018;19(9):2549.

65. Williams JW, Giannarelli C, Rahman A, Randolph GJ, Kovacic JC. Macrophage biology, classification, and phenotype in cardiovascular disease: JACC macrophage in CVD series (part 1). Journal of the American College of Cardiology. 2018;72(18):2166-80.

66. Lehner C, Spitzer G, Gehwolf R, Wagner A, Weissenbacher N, Deininger C, et al. Tenophages: a novel macrophage-like tendon cell population expressing CX3CL1 and CX3CR1. Disease models & mechanisms. 2019;12(12):dmm041384.

67. Tarafder S, Chen E, Jun Y, Kao K, Sim KH, Back J, et al. Tendon stem/progenitor cells regulate inflammation in tendon healing via JNK and STAT3 signaling. The FASEB journal. 2017;31(9):3991.

68. Lu J, Chen H, Lyu K, Jiang L, Chen Y, Long L, et al. The functions and mechanisms of tendon stem/progenitor cells in tendon healing. Stem Cells International. 2023;2023(1):1258024.

69. Stolk M, Klatte-Schulz F, Schmock A, Minkwitz S, Wildemann B, Seifert M. New insights into tenocyte-immune cell interplay in an in vitro model of inflammation. Scientific reports. 2017;7(1):9801.

70. Dakin SG, Martinez FO, Yapp C, Wells G, Oppermann U, Dean BJ, et al. Inflammation activation and resolution in human tendon disease. Science translational medicine. 2015;7(311):311ra173-311ra173.

71. Tang C, Chen Y, Huang J, Zhao K, Chen X, Yin Z, et al. The roles of inflammatory mediators and immunocytes in tendinopathy. Journal of Orthopaedic Translation. 2018;14:23-33.

72. Akbar M, MacDonald L, Crowe LA, Carlberg K, Kurowska-Stolarska M, Ståhl PL, et al. Single cell and spatial transcriptomics in human tendon disease indicate dysregulated immune homeostasis. Annals of the Rheumatic Diseases. 2021;80(11):1494-7.

73. Stańczak M, Kacprzak B, Gawda P. Tendon cell biology: Effect of mechanical loading. Cell Physiol Biochem. 2024;58(6):677-701.

74. De Bastiani G, de La Côrte FD, Brass KE, Kommers GD, Denoix JM. Association of ultrasound and anatomopathologic findings of equine metacarpophalangeal lesions. Journal of Equine Veterinary Science. 2014;34(10):1218-25.

75. Jones DL, Hallström GF, Jiang X, Locke RC, Evans MK, Bonnevie ED, et al. Mechanoepigenetic regulation of extracellular matrix homeostasis via Yap and Taz. Proceedings of the National Academy of Sciences. 2023;120(22):e2211947120.

76. Piccolo S, Dupont S, Cordenonsi M. The biology of YAP/TAZ: hippo signaling and beyond. Physiological reviews. 2014;94(4):1287-312.

77. Gomez-Florit M, Labrador-Rached CJ, Domingues RM, Gomes ME. The tendon microenvironment: Engineered in vitro models to study cellular crosstalk. Advanced Drug Delivery Reviews. 2022;185:114299.

78. Peserico A, Barboni B, Russo V, Bernabò N, El Khatib M, Prencipe G, et al. Mammal comparative tendon biology: advances in regulatory mechanisms through a computational modeling. Frontiers in Veterinary Science. 2023;10:1175346.

79. Hou J, Yang R, Vuong I, Li F, Kong J, Mao H-Q. Biomaterials strategies to balance inflammation and tenogenesis for tendon repair. Acta biomaterialia. 2021;130:1-16.

80. Ackerman JE, Best KT, Muscat SN, Loiselle AE. Metabolic regulation of tendon inflammation and healing following injury. Current rheumatology reports. 2021;23:1-9.

81. Chan K-M, Fu S-C. Anti-inflammatory management for tendon injuries-friends or foes? BMC Sports Science, Medicine and Rehabilitation. 2009;1:1-3.

82. Crosio G, Huang A. Innate and adaptive immune cells implicated in tendon healing and disease. European cells & materials. 2022;43:39.

83. Marques-Smith P, Kallerud AS, Johansen GM, Boysen P, Jacobsen AM, Reitan KM, et al. Is clinical effect of autologous conditioned serum in spontaneously occurring equine articular lameness related to ACS cytokine profile? BMC veterinary research. 2020;16:1-9.

84. Tokawa PKA, Brossi PM, Baccarin RYA. Autologous conditioned serum in equine and human orthopedic therapy: A systematic review. Research in Veterinary Science. 2022;146:34-52.

85. Velloso Alvarez A, Boone LH, Pondugula SR, Caldwell F, Wooldridge AA. Effects of autologous conditioned serum, autologous protein solution, and triamcinolone on inflammatory and catabolic gene expression in equine cartilage and synovial explants treated with IL-1β in co-culture. Frontiers in veterinary science. 2020;7:323.

86. Majewski M, Ochsner PE, Liu F, Flückiger R, Evans CH. Accelerated healing of the rat Achilles tendon in response to autologous conditioned serum. The American journal of sports medicine. 2009;37(11):2117-25.

87. Genç E, Beytemur O, Yuksel S, Eren Y, Çağlar A, Küçükyıldırım BO, et al. Investigation of the biomechanical and histopathological effects of autologous conditioned serum on healing of Achilles tendon. Acta orthopaedica et traumatologica turcica. 2018;52(3):226-31.

88. von Wehren L, Pokorny K, Blanke F, Sailer J, Majewski M. Injection with autologous conditioned serum has better clinical results than eccentric training for chronic Achilles tendinopathy. Knee Surgery, Sports Traumatology, Arthroscopy. 2019;27:2744-53.

89. Geburek F, Lietzau M, Beineke A, Rohn K, Stadler PM, therapy. Effect of a single injection of autologous conditioned serum (ACS) on tendon healing in equine naturally occurring tendinopathies. Stem cell research. 2015;6:1-14.

90. Gaesser AM, Underwood C, Linardi RL, Even KM, Reef VB, Shetye SS, et al. Evaluation of autologous protein solution injection for treatment of superficial digital flexor tendonitis in an equine model. Frontiers in Veterinary Science. 2021;8:697551.

91. Knott LE, Fonseca‐Martinez BA, O'Connor AM, Goodrich LR, McIlwraith CW, Colbath AC. Current use of biologic therapies for musculoskeletal disease: a survey of board‐certified equine specialists. Veterinary Surgery. 2022;51(4):557-67.

92. Nasirzade J, Kargarpour Z, Hasannia S, Strauss FJ, Gruber R. Platelet‐rich fibrin elicits an anti‐inflammatory response in macrophages in vitro. Journal of periodontology. 2020;91(2):244-52.

93. Zhang J, Yin C, Zhao Q, Zhao Z, Wang J, Miron RJ, et al. Anti-inflammation effects of injectable platelet-rich fibrin via macrophages and dendritic cells. J Biomed Mater Res A. 2020;108(1):61-8.

94. Tognoloni A, Bartolini D, Pepe M, Di Meo A, Porcellato I, Guidoni K, et al. Platelets rich plasma increases antioxidant defenses of tenocytes via Nrf2 signal pathway. International Journal of Molecular Sciences. 2023;24(17):13299.

95. Shi Y, Kang X, Wang Y, Bian X, He G, Zhou M, et al. Exosomes derived from bone marrow stromal cells (BMSCs) enhance tendon-bone healing by regulating macrophage polarization. Medical science monitor: international medical journal of experimental and clinical research. 2020;26:e923328-1.

96. Zhang M, Liu H, Cui Q, Han P, Yang S, Shi M, et al. Tendon stem cell-derived exosomes regulate inflammation and promote the high-quality healing of injured tendon. Stem cell research & therapy. 2020;11(1):1-15.

97. Xu T, Lin Y, Yu X, Jiang G, Wang J, Xu K, et al. Comparative effects of exosomes and ectosomes isolated from adipose-derived mesenchymal stem cells on achilles tendinopathy in a rat model. The American journal of sports medicine. 2022;50(10):2740-52.

98. Shi Z, Wang Q, Jiang D. Extracellular vesicles from bone marrow-derived multipotent mesenchymal stromal cells regulate inflammation and enhance tendon healing. Journal of translational medicine. 2019;17:1-12.

99. Lu V, Tennyson M, Zhang J, Khan W. Mesenchymal stem cell-derived extracellular vesicles in tendon and ligament repair—A systematic review of in vivo studies. Cells. 2021;10(10):2553.

100. Chen W, Huang Y, Han J, Yu L, Li Y, Lu Z, et al. Immunomodulatory effects of mesenchymal stromal cells-derived exosome. Immunologic research. 2016;64:831-40.

101. Shen H, Yoneda S, Abu‐Amer Y, Guilak F, Gelberman RH. Stem cell‐derived extracellular vesicles attenuate the early inflammatory response after tendon injury and repair. Journal of Orthopaedic Research®. 2020;38(1):117-27.

102. Kim M, Shin DI, Choi BH, Min B-H. Exosomes from IL-1β-primed mesenchymal stem cells inhibited IL-1β-and TNF-α-mediated inflammatory responses in osteoarthritic SW982 cells. Tissue Engineering and Regenerative Medicine. 2021:1-12.

103. Cooper HE. Equine Bone Marrow Mesenchymal Stem Cell-Derived Extracellular Vesicle Anti-Inflammatory Properties on Tenocyte-Macrophage In-Vitro Co-Cultures: The Ohio State University; 2024.

104. Quam VG, Belacic ZA, Long S, Rice HC, Dhar MS, Durgam S. Equine bone marrow MSC‐derived extracellular vesicles mitigate the inflammatory effects of interleukin‐1β on navicular tissues in vitro. Equine Veterinary Journal. 2025;57(1):232-42.

105. Quintero D, Perucca Orfei C, Kaplan LD, de Girolamo L, Best TM, Kouroupis D. The roles and therapeutic potentialof mesenchymal stem/stromal cells and their extracellular vesicles in tendinopathies. Frontiers in bioengineering and biotechnology. 2023;11:1040762.

106. Shen H, Lane RA. Extracellular vesicles from primed adipose-derived stem cells enhance Achilles tendon repair by reducing inflammation and promoting intrinsic healing. Stem Cells. 2023;41(6):617-27.

107. Bosch G, Moleman M, Barneveld A, van Weeren PR, Van Schie H. The effect of platelet‐rich plasma on the neovascularization of surgically created equine superficial digital flexor tendon lesions. Scandinavian journal of medicine & science in sports. 2011;21(4):554-61.

108. Brossi PM, Moreira JJ, Machado TS, Baccarin RY. Platelet-rich plasma in orthopedic therapy: a comparative systematic review of clinical and experimental data in equine and human musculoskeletal lesions. BMC veterinary research. 2015;11:1-17.

109. Beerts C, Suls M, Broeckx SY, Seys B, Vandenberghe A, Declercq J, et al. Tenogenically induced allogeneic peripheral blood mesenchymal stem cells in allogeneic platelet-rich plasma: 2-year follow-up after tendon or ligament treatment in horses. Frontiers in Veterinary Science. 2017;4:158.

110. Depuydt E, Broeckx SY, Van Hecke L, Chiers K, Van Brantegem L, Van Schie H, et al. The evaluation of equine allogeneic tenogenic primed mesenchymal stem cells in a surgically induced superficial digital flexor tendon lesion model. Frontiers in veterinary science. 2021;8:641441.

111. Trouski FF, Parham A. Exosomes Derived from Mesenchymal Stem Cells in the Treatment of Animal Tendon Injuries: A Review on Their Isolation and Application. Iranian Journal of Veterinary Medicine. 2021;15(3).

112. Depuydt E, Broeckx SY, Chiers K, Patruno M, Da Dalt L, Duchateau L, et al. Cellular and humoral immunogenicity investigation of single and repeated allogeneic tenogenic primed mesenchymal stem cell treatments in horses suffering from tendon injuries. Frontiers in Veterinary Science. 2022;8:789293.

113. Burk J, Wittenberg-Voges L, Schubert S, Horstmeier C, Brehm W, Geburek F. Treatment of naturally occurring tendon disease with allogeneic multipotent mesenchymal stromal cells: a randomized, controlled, triple-blinded pilot study in horses. Cells. 2023;12(21):2513.

114. Salz RO, Elliott CR, Zuffa T, Bennet ED, Ahern BJ. Treatment of racehorse superficial digital flexor tendonitis: a comparison of stem cell treatments to controlled exercise rehabilitation in 213 cases. Equine Veterinary Journal. 2023;55(6):979-87.

115. Zhu Y, Yan J, Zhang H, Cui G. Bone marrow mesenchymal stem cell-derived exosomes: A novel therapeutic agent for tendon-bone healing. International Journal of Molecular Medicine. 2023;52(6):121.

116. Carlier S, Depuydt E, Suls M, Bocqué C, Thys J, Vandenberghe A, et al. Equine allogeneic tenogenic primed mesenchymal stem cells: a clinical field study in horses suffering from naturally occurring superficial digital flexor tendon and suspensory ligament injuries. Equine Veterinary Journal. 2024;56(5):924-35.

117. Dejnek M, Witkowski J, Moreira H, Płaczkowska S, Morasiewicz P, Reichert P, et al. Content of blood cell components, inflammatory cytokines and growth factors in autologous platelet-rich plasma obtained by various methods. World Journal of Orthopedics. 2022;13(6):587.

118. Radtke AV, Goodale MB, Fortier LA. Platelet and leukocyte concentration in equine autologous conditioned plasma are inversely distributed by layer and are not affected by centrifugation rate. Frontiers in Veterinary Science. 2020;7:173.

119. Georg R, Maria C, Gisela A, Bianca C. Autologous conditioned plasma as therapy of tendon and ligament lesions in seven horses. Journal of Veterinary Science. 2010;11(2):173-5.

120. Estrada RJ, van Weeren R, van de Lest CH, Boere J, Reyes M, Ionita J-C, et al. Effects of Autologous Conditioned Plasma®(ACP) on the healing of surgically induced core lesions in equine superficial digital flexor tendon. Pferdeheilkunde. 2014;30(6):633-42.

121. McCarrel TM, Minas T, Fortier LA. Optimization of leukocyte concentration in platelet-rich plasma for the treatment of tendinopathy. JBJS. 2012;94(19):e143.

122. Yan R, Gu Y, Ran J, Hu Y, Zheng Z, Zeng M, et al. Intratendon delivery of leukocyte-poor platelet-rich plasma improves healing compared with leukocyte-rich platelet-rich plasma in a rabbit Achilles tendinopathy model. The American journal of sports medicine. 2017;45(8):1909-20.

123. Romero A, Barrachina L, Ranera B, Remacha A, Moreno B, De Blas I, et al. Comparison of autologous bone marrow and adipose tissue derived mesenchymal stem cells, and platelet rich plasma, for treating surgically induced lesions of the equine superficial digital flexor tendon. The Veterinary Journal. 2017;224:76-84.

124. Zwittnig K, Kirnbauer B, Jakse N, Schlenke P, Mischak I, Ghanaati S, et al. Growth factor release within liquid and solid PRF. Journal of clinical medicine. 2022;11(17):5070.

125. Zuleika P, Saleh I, Murti K, Liberty IA, Surono A. Platelet-rich Fibrin: A Systematic Review of Its Action. Journal of Nature Science of Medicine. 2024;7(4):242-52.

126. Farmani AR, Nekoofar MH, Ebrahimi Barough S, Azami M, Rezaei N, Najafipour S, et al. Application of platelet rich fibrin in tissue engineering: focus on bone regeneration. J Platelets. 2021;32(2):183-8.

127. Najeb M, Samy A, Rizk A, Mosbah E, Karrouf G. Clinical benefits of early-stage autologous conditioned serum and injectable platelet-rich fibrin on healing superficial digital flexor tendonitis in donkeys. Irish Veterinary Journal. 2025;78(1):1-14.

128. Montes A, Mauro A, Cerveró-Varona A, Prencipe G, El Khatib M, Tosi U, et al., editors. NOVEL STEM CELL STRATEGIES FOR TENDON REGENERATIVE MEDICINE. Orthopaedic Proceedings; 2024: Bone & Joint.

129. Smith RK. Stem cell therapies for equine tendinopathy. Ciencias Veterinarias. 2019;37(3):20-1.

130. Carlier S, Depuydt E, Van Hecke L, Martens A, Saunders J, Spaas JH. Safety assessment of equine allogeneic tenogenic primed mesenchymal stem cells in horses with naturally occurring tendon and ligament injuries. Frontiers in Veterinary Science. 2024;11:1282697.

131. Cequier A, Vazquez F, Barrachina L, Romero A, Rodellar C, Vitoria A, et al. Humoral immune response against allogeneic equine mesenchymal stem cells (MSCs) mediated by the major histocompatibility complex (MHC): an issue to take into account for the safety and efficacy of treatment with MSCs. 2019.

132. Cequier A, Vázquez FJ, Vitoria A, Bernad E, Fuente S, Serrano MB, et al. The systemic cellular immune response against allogeneic mesenchymal stem cells is influenced by inflammation, differentiation and MHC compatibility: in vivo study in the horse. Frontiers in Veterinary Science. 2024;11:1391872.

133. Li Y, Wu T, Liu S. Identification and distinction of tenocytes and tendon-derived stem cells. Frontiers in Cell and Developmental Biology. 2021;9:629515.

134. He W, Jiang C, Zhou P, Hu X, Gu X, Zhang S. Role of tendon-derived stem cells in tendon and ligament repair: focus on tissue engineer. Frontiers in Bioengineering and Biotechnology. 2024;12:1357696.

135. Guest D, Smith M, Allen W. Equine embryonic stem‐like cells and mesenchymal stromal cells have different survival rates and migration patterns following their injection into damaged superficial digital flexor tendon. Equine veterinary journal. 2010;42(7):636-42.

136. Spees JL, Lee RH, Gregory CA. Mechanisms of mesenchymal stem/stromal cell function. Stem cell research & therapy. 2016;7:1-13.

137. Vizoso FJ, Eiro N, Cid S, Schneider J, Perez-Fernandez R. Mesenchymal stem cell secretome: toward cell-free therapeutic strategies in regenerative medicine. International journal of molecular sciences. 2017;18(9):1852.

138. Lee B-C, Kang I, Yu K-R. Therapeutic features and updated clinical trials of mesenchymal stem cell (MSC)-derived exosomes. Journal of clinical medicine. 2021;10(4):711.

139. Wang Y, He G, Guo Y, Tang H, Shi Y, Bian X, et al. Exosomes from tendon stem cells promote injury tendon healing through balancing synthesis and degradation of the tendon extracellular matrix. Journal of Cellular and Molecular Medicine. 2019;23(8):5475-85.

140. Yu H, Cheng J, Shi W, Ren B, Zhao F, Shi Y, et al. Bone marrow mesenchymal stem cell-derived exosomes promote tendon regeneration by facilitating the proliferation and migration of endogenous tendon stem/progenitor cells. Acta biomaterialia. 2020;106:328-41.

141. Yao Z, Li J, Xiong H, Cui H, Ning J, Wang S, et al. MicroRNA engineered umbilical cord stem cell-derived exosomes direct tendon regeneration by mTOR signaling. Journal of Nanobiotechnology. 2021;19(1):169.

142. Aydın EY, Aşık M, Aydın HM, Çay N, Gümüşkaya B, Çağlayan A, et al. The co-use of stromal vascular fraction and bone marrow concentrate for tendon healing. Current stem cell research & therapy. 2023;18(8):1150-9.

143. Polly SS, Nichols AE, Donnini E, Inman DJ, Scott TJ, Apple SM, et al. Adipose‐derived stromal vascular fraction and cultured stromal cells as trophic mediators for tendon healing. Journal of Orthopaedic Research®. 2019;37(6):1429-39.

144. Senesi L, De Francesco F, Marchesini A, Pangrazi PP, Bertolini M, Riccio V, et al. Efficacy of adipose-derived mesenchymal stem cells and stromal vascular fraction alone and combined to biomaterials in tendinopathy or tendon injury: Systematic review of current concepts. Medicina. 2023;59(2):273.

145. Tyrnenopoulou P, Diakakis N, Angelopoulou S, Pyrros A, Mparous E, Koliakos G, et al. Clinical application of adipose-derived stromal vascular fraction in 3 Thoroughbred horses with superficial digital flexor tendonitis: Case Report. Journal of the Hellenic Veterinary Medical Society. 2015;66(4):215-22.

146. Fortier LA. Equine bone marrow aspirate concentrate. Veterinary Clinics: Equine Practice. 2023;39(3):453-9.

147. Lana JF, da Fonseca LF, Azzini G, Santos G, Braga M, Cardoso Junior AM, et al. Bone marrow aspirate matrix: a convenient ally in regenerative medicine. International Journal of Molecular Sciences. 2021;22(5):2762.

148. Long S, Maleas G, Belacic ZA, Quam VG, Durgam S. Equine bone marrow aspirate and bone marrow aspirate concentrate are enriched with interleukin-1 receptor antagonist protein. American Journal of Veterinary Research. 2025;86(5).

149. Crovace A, Lacitignola L, Rossi G, Francioso E. Histological and immunohistochemical evaluation of autologous cultured bone marrow mesenchymal stem cells and bone marrow mononucleated cells in collagenase‐induced tendinitis of equine superficial digital flexor tendon. Veterinary Medicine International. 2010;2010(1):250978.

150. Russell J, Russell T, Vasey J, Hall M. Autologous bone marrow aspirate for treatment of superficial digital flexor tendonitis in 105 racehorses. Veterinary Record. 2016;179(3):69-.

151. Yuksel S, Guleç MA, Gultekin MZ, Adanır O, Caglar A, Beytemur O, et al. Comparison of the early period effects of bone marrow-derived mesenchymal stem cells and platelet-rich plasma on the Achilles tendon ruptures in rats. Connective Tissue Research. 2016;57(5):360-73.

152. Veronesi F, Pagani S, Torricelli P, Filardo G, Cavallo C, Grigolo B, et al. PRP and MSCs on tenocytes artificial wound healing: an in vitro study comparing fresh and frozen PRP. 2018.

153. Han L, Fang W, Jin B, Xu S, Zheng X, Hu Y. Enhancement of tendon-bone healing after rotator cuff injuries using combined therapy with mesenchymal stem cells and platelet rich plasma. European Review for Medical & Pharmacological Sciences. 2019;23(20).

154. Soler R, Rodas G, Rius-Tarruella J, Alomar X, Balius R, Ruíz-Cotorro Á, et al. Safety and Efficacy of Bone Marrow–Derived Mesenchymal Stem Cells for Chronic Patellar Tendinopathy (with Gap> 3 Mm) in Patients: 12-Month Follow-up Results of a Phase 1/2 Clinical Trial. Orthopaedic Journal of Sports Medicine. 2023;11(9):23259671231184400.

155. Ortega-Cebrián S, Soler-Rich R, Orozco L, Rodas G. Evaluation of Patellar Tendon Structural Changes following Biological Treatments: Secondary Analysis of Double-Blinded Clinical Trial of Bone Marrow Mesenchymal Stromal Cells and Leukocyte-Poor Platelet-Rich Plasma. Biomedicines. 2024;12(7):1599.

156. Goulian AJ, Goldstein B, Saad MA. Advancements in Regenerative Therapies for Orthopedics: A Comprehensive Review of Platelet-Rich Plasma, Mesenchymal Stem Cells, Peptide Therapies, and Biomimetic Applications. Journal of Clinical Medicine. 2025;14(6):2061.

157. Carvalho AM, Badial PR, Álvarez LEC, Yamada ALM, Borges AS, Deffune E, et al. Equine tendonitis therapy using mesenchymal stem cells and platelet concentrates: a randomized controlled trial. Stem cell research & therapy. 2013;4:1-13.

158. Ricco' S, Renzi S, Del Bue M, Conti V, Merli E, Ramoni R, et al. Allogeneic adipose tissue-derived mesenchymal stem cells in combination with platelet rich plasma are safe and effective in the therapy of superficial digital flexor tendonitis in the horse. International journal of immunopathology and pharmacology. 2013;26(1_suppl):61-8.

159. Melotti L, Carolo A, Elshazly N, Boesso F, Da Dalt L, Gabai G, et al. Case Report: Repeated Intralesional Injections of Autologous Mesenchymal Stem Cells Combined With Platelet-Rich Plasma for Superficial Digital Flexor Tendon Healing in a Show Jumping Horse. Frontiers in Veterinary Science. 2022;9:843131.

160. Smith RK, Schramme MC. Clinical Updates on Superficial Digital Flexor Tendon Injuries: Insights on Diagnostic and Therapeutic Advancements. Veterinary Clinics: Equine Practice. 2025;41(2):279-98.

161. Lang HM, Schnabel LV, Cassano JM, Fortier LA. Effect of needle diameter on the viability of equine bone marrow derived mesenchymal stem cells. Veterinary Surgery. 2017;46(5):731-7.

162. Miron RJ, Gruber R, Farshidfar N, Sculean A, Zhang Y. Ten years of injectable platelet‐rich fibrin. Periodontology 2000. 2024;94(1):92-113.

163. Oreff GL, Fenu M, Vogl C, Ribitsch I, Jenner F. Species variations in tenocytes’ response to inflammation require careful selection of animal models for tendon research. Scientific Reports. 2021;11(1):12451.

164. Beaumont RE, Smith EJ, David C, Paterson YZ, Faull E, Guest DJ. Equine adult, fetal and ESC-tenocytes have differential migratory, proliferative and gene expression responses to factors upregulated in the injured tendon. Cells & Development. 2025;181:204003.

165. Shojaee A. Equine tendon mechanical behaviour: Prospects for repair and regeneration applications. Veterinary Medicine Science. 2023;9(5):2053-69.

166. Ellis IM, Schnabel LV, Berglund AK. Defining the profile: characterizing cytokines in tendon injury to improve clinical therapy. Journal of immunology and regenerative medicine. 2022;16:100059.

167. Lotfy A, AboQuella NM, Wang H. Mesenchymal stromal/stem cell (MSC)-derived exosomes in clinical trials. Stem Cell Research & Therapy. 2023;14(1):66.

168. Zhang Y, Ju W, Zhang H, Mengyun L, Shen W, Chen X. Mechanisms and therapeutic prospects of mesenchymal stem cells-derived exosomes for tendinopathy. Stem Cell Research & Therapy. 2023;14(1):307.

169. Zou J, Yang W, Cui W, Li C, Ma C, Ji X, et al. Therapeutic potential and mechanisms of mesenchymal stem cell-derived exosomes as bioactive materials in tendon–bone healing. Journal of nanobiotechnology. 2023;21(1):14.

170. Roszkowski S. Therapeutic potential of mesenchymal stem cell-derived exosomes for regenerative medicine applications. Clinical and Experimental Medicine. 2024;24(1):46.

171. Al Naem M, Bourebaba L, Kucharczyk K, Röcken M, Marycz K. Therapeutic mesenchymal stromal stem cells: Isolation, characterization and role in equine regenerative medicine and metabolic disorders. Stem cell reviews and reports. 2020;16(2):301-22.

**Figure 1.** Overview of key players and events during the tendon repair process after injury follows three overlapping stages: inflammation, proliferation, and remodeling. Each stage is characterized by specific cellular and molecular events that drive the healing process. Abbreviations: TNF-α, tumor necrosis factor; VEGF, vascular endothelial growth factor; PDGF, platelet-derived growth factor; FGF, fibroblast growth factor, TGF-β, transforming growth factor beta; CTGF, connective tissue growth factor; IGF-1, insulin-like growth factor-1; RBC, red blood cell; Scx, scleraxis **(79)**

**Figure 2.** Initiation of a type 1 and type 2 immune response and the subsequent macrophage phenotype spectrum, including M1and M2, are not fixed; macrophages can transition between them in response to different environmental signals. Abbreviations: DAMP, damage-associated molecular patterns; T-cells, T lymphocyte; Th-cells, T helper lymphocyte; Treg, T regulatory lymphocyte; Mϕ, macrophage; TNF-α, tumour necrosis factor alpha; IFN-γ, interferon gamma; IL, interleukin; IL-1Ra, interleukin 1 receptor antagonist **(22)**.

**Figure 3.** Schematic representation of macrophage polarization within the tendon microenvironment. Uncommitted macrophages can polarize into either M1 or M2 phenotypes in response to external cues. Persistent pro-inflammatory signals such as IL-1 and DAMPs drive macrophages toward the M1 state through NF-κB pathways, maintaining an inflammatory environment. In contrast, tendon-derived CD146⁺ progenitor cells contribute to M2 polarization by secreting anti-inflammatory mediators (IL-10, TIMP-3) and releasing exosomes enriched with regulatory microRNAs and proteins. Inflammation-resolving pathways mediated by glucocorticoid receptor activation further support M2 polarization.

**Figure 4.** Illustration of exosome biogenesis, structure, and functional role in tendon healing. MSCs-derived exosomes carry regulatory proteins and RNAs, and upon delivery to recipient cells, they reduce inflammatory cell recruitment, shift macrophages from the M1 to M2 phenotype, and suppress pro-inflammatory cytokines (e.g., TNF-α, IL-1β) while enhancing anti-inflammatory mediators (e.g., IL-10, TGF-β) **(169)**.

| **Table 1.** Temporal progression of tendon healing: molecular mediators, clinical correlates, and targeted therapeutics **(10).** | | | | | |
| --- | --- | --- | --- | --- | --- |
| **Repair phase** | **Mediators** | **Activity** | **Clinical signs** | **Treatment strategy** | **Treatment choices** |
| **Acute Inflammatory phase (1-2 weeks)** | IL-1β  TNF-α  IFN-γ  IL-6  IL-12 | Inflammatory mediators regulate leukocyte and fibroblast migration to the injury site.  Release of proteolytic enzymes (MMP-1) for the removal of damaged tissue, but tends to be indiscriminate and includes the removal of adjacent healthy tissue.  Express other GF  Angiogenesis | Pain upon palpation  Heat  Tendon swelling  Lameness | Reduce (but not eliminate) inflammation | Physical cold therapies  Systemic or peritendinous specific anti-inflammatory medication |
| **Subacute Proliferative**  **Phase**  **(3-16 weeks)** | IL-10  IL-1Ra  IL4  IL13  IL33 | Anti-inflammatory and inflammation-modulating mediators  Fibroblast proliferation  Synthesis of Type III Collagen  Stimulates interactions of ECM (increased glycosaminoglycan content and ultra-structurally universally small fibrils) | Reduction or absence of lameness  Resolution of signs of inflammation  The tendon is still palpably enlarged and soft | Promote the regeneration of a functionally normal tendon  Optimize the organization of scar tissue. | Early controlled mobilization with ultrasound monitoring  Intralesional treatment, for example, biological (growth factors (e.g., PRP); mesenchymal stem cells) |
| **Chronic Remodeling phase (> 16 weeks)** |  | Termination of cell proliferation  Collagen type I synthesis  ECM remodeling | Tendon size decreases  Tendon less pliable | Promote remodelling  Prevent re-injury | Controlled ascending exercise regime with ultrasound monitoring |
| Abbreviations: IGF-1, insulin-like growth factor 1; TGF-β, transforming growth factor beta; PDGF, platelet-derived growth factor; bFGF, basic fibroblast growth factor; VEGF, vascular endothelial growth factor; MMP-1, matrix metalloproteinase 1; ECM, extracellular matrix; PRP, platelet-rich plasma; IL, interleukin; IL-1Ra, interleukin 1 receptor antagonist protein; TNF-α, tumor necrosis factor alpha; IFN-γ, interferon gamma. | | | | | |

| Table 2. Practical considerations for regenerative biologics in equine SDF tendonitis. | | | | | |
| --- | --- | --- | --- | --- | --- |
| Therapy | Active component | Timing of Use /  Case Selection | Preparation / Availability & Origin | Administration | Considerations / Risks |
| Autologous conditioned serum (ACS)  **(89)** | IL-1Ra | Acute inflammatory phase, and early proliferative | Blood incubation at 37°C in a glass bead tube: 10 mL for 6–9 h, 60 mL for 18-24 h  Centrifugation: at 4000 rpm × 10 min  Filtration through a 0.22 μm sterile filter  Used fresh or stored frozen | Injected every 7–10 days × 2–3 doses | Over-incubation induces hemolysis & pro-inflammatory factors (red tint) Repeated freeze–thaw (>3) may reduce the effect |
| Autologous protein solution (APS)  **(90)** | Platelets, Growth factors (GFs), &  IL-1Ra |  | Two-step, stall-side centrifugation:  Platelet concentration: 3200 rpm × 15 min with acid citrate dextrose (ACD) anticoagulant.  Cytokine enrichment: Buffy coat + plasma centrifuged with polyacrylamide beads (2–3 min) to concentrate IL-1Ra and GFs | Single intralesional injection |  |
| Exosomes  **(170)** | MicroRNAs  Proteins |  | Isolated from MSCs-conditioned media  Commercially available (allogenic)  Higher potency when derived from MSCs primed toward tenogenesis or with pro-inflammatory cytokines (e.g., IL-1β, TNF-α) | Combine with carriers like PRP or fibrin to prevent rapid clearance | Poor retention if injected alone; best used as an adjunct in multimodal therapy |
| Platelet-rich plasma (PRP)  **(16)** | Platelets, GFs | Early proliferative phase  Acute/subacute lesions with active healing  Not suitable for chronic fibrotic tissue | Anticoagulant (ACD or similar); double-spin (3200 rpm × 15 min, then 3500 rpm × 10 min). Activate with calcium chloride or thrombin before use | Effect lasts 3–7 days  Requires frequent reinjection | More complex preparation than PRF  Chemical additives may alter its function |
| Autologous conditioned plasma (ACP) **(117)** | Platelets, GFs |  | Point-of-care double-syringe kit  15–60 mL blood + ACD-A; centrifuge ~1500 rpm × 5 min; yield 2–7 mL ACP |  | Faster preparation than PRP, but typically lower platelet/ GFs content |
| Injectable platelet-rich fibrin (I-PRF)  **(162)** | GFs & Fibrin network |  | Immediate centrifugation at 700 rpm × 3 min in plastic plain tubes  Rapid, stall-side preparation | Sustained GFs release lasts over 2 weeks | Use an 18-G needle  Must be injected within 5–10 min to prevent clotting |
| Mesenchymal stem cells (MSCs)  **(171)** | Stem cells for differentiation & paracrine signaling | Proliferative to early remodeling phases  Moderate to severe lesions | Autologous (bone marrow, adipose) or commercial allogeneic sources | Used with a ≥20G needle to protect cells  Injected alone or in multimodal therapy | Risk of immune rejection (allogeneic)  Loss of viability with mishandling  Potential uncontrolled differentiation |
| Stromal vascular fraction (SVF)  **(143)** | Mixed cell population (MSCs, hematopoietic, endothelial, etc.) |  | Harvest adipose tissue, enzymatically or mechanically digest + centrifuge 3000rpm/5 min to isolate SVF |  | Single-step  same-day use  lower cost compared to autogenous MSCs |
| Bone marrow aspirate concentrates (BMAC)  **(146)** | MSCs, hematopoietic, platelets, GFs |  | Bone marrow aspirate into anticoagulant → centrifuge ~3000 rpm × 10–20 min (double-syringe or lab system) |  | Lower MSCs concentration vs. cultured MSCs |

**
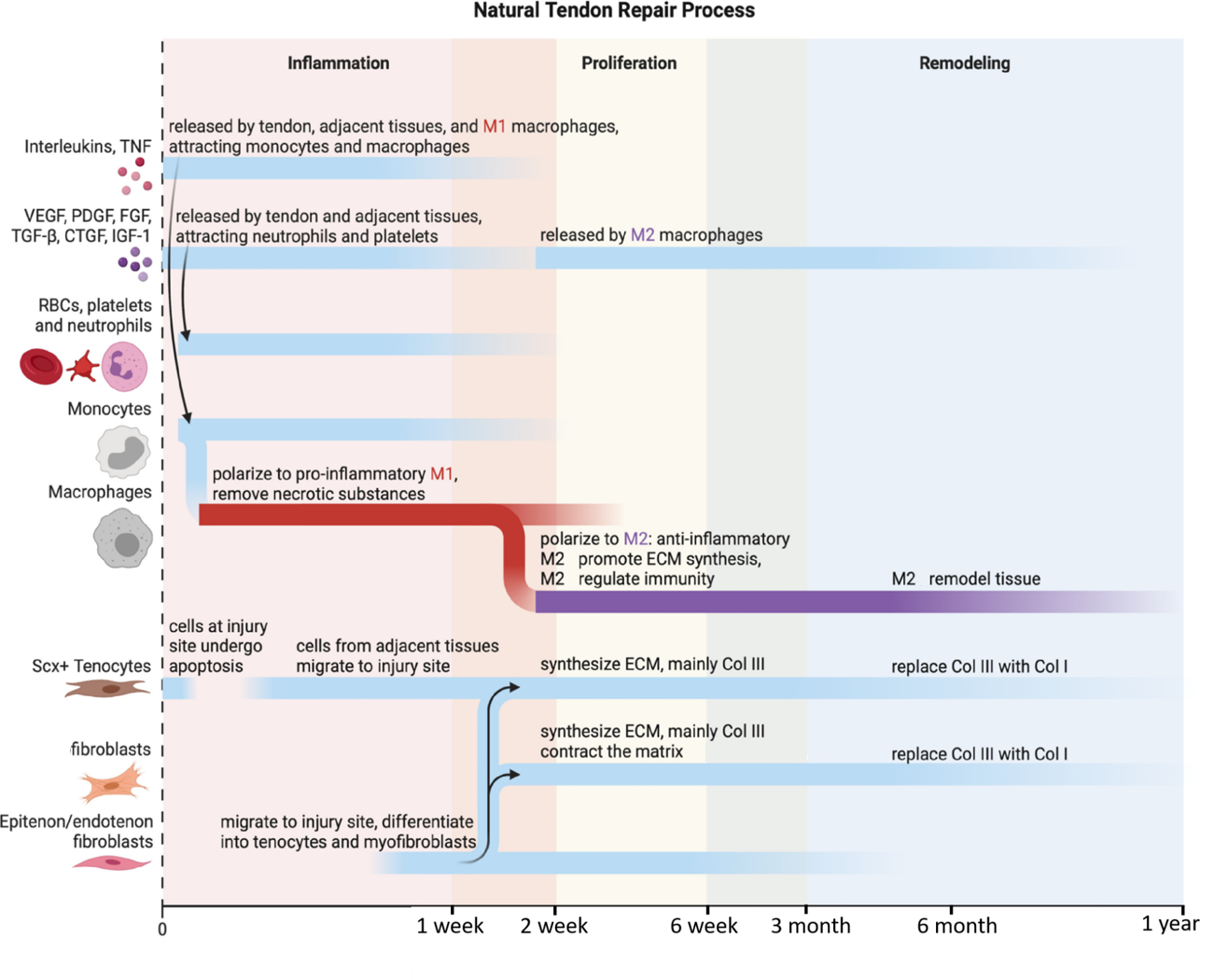
**

**Figure 1.** Overview of key players and events during the tendon repair process after injury follows three overlapping stages: inflammation, proliferation, and remodeling. Each stage is characterized by specific cellular and molecular events that drive the healing process. Abbreviations: TNF-α, tumor necrosis factor; VEGF, vascular endothelial growth factor; PDGF, platelet-derived growth factor; FGF, fibroblast growth factor; TGF-β, transforming growth factor beta; CTGF, connective tissue growth factor; IGF-1, insulin-like growth factor-1; RBC, red blood cell; Scx, scleraxis **(79)**.

**
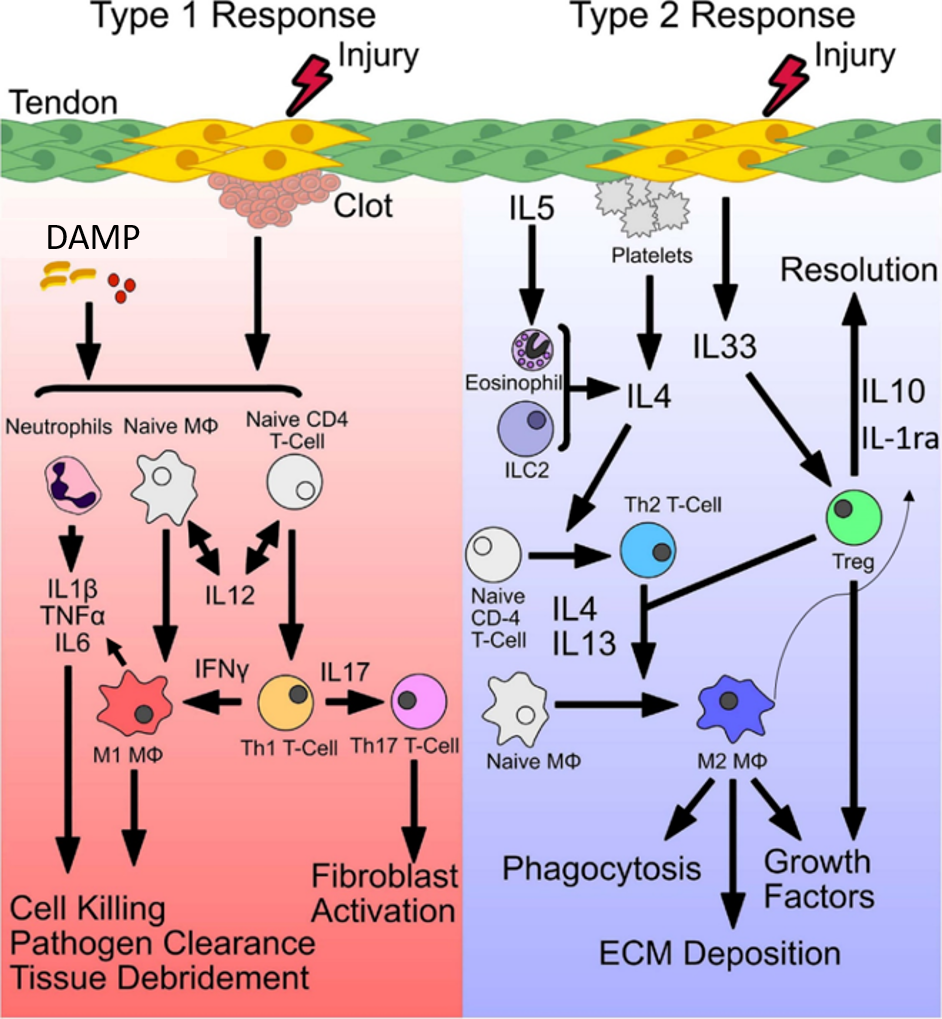
**

**Figure 2.** Initiation of a type 1 and type 2 immune response and the subsequent macrophage phenotype spectrum, including M1and M2, are not fixed; macrophages can transition between them in response to different environmental signals. Abbreviations: DAMP, damage-associated molecular patterns; T-cells, T lymphocyte; Th-cells, T helper lymphocyte; Treg, T regulatory lymphocyte; Mϕ, macrophage; TNF-α, tumor necrosis factor alpha; IFN-γ, interferon gamma; IL, interleukin; IL-1Ra, interleukin 1 receptor antagonist **(22)**.

**
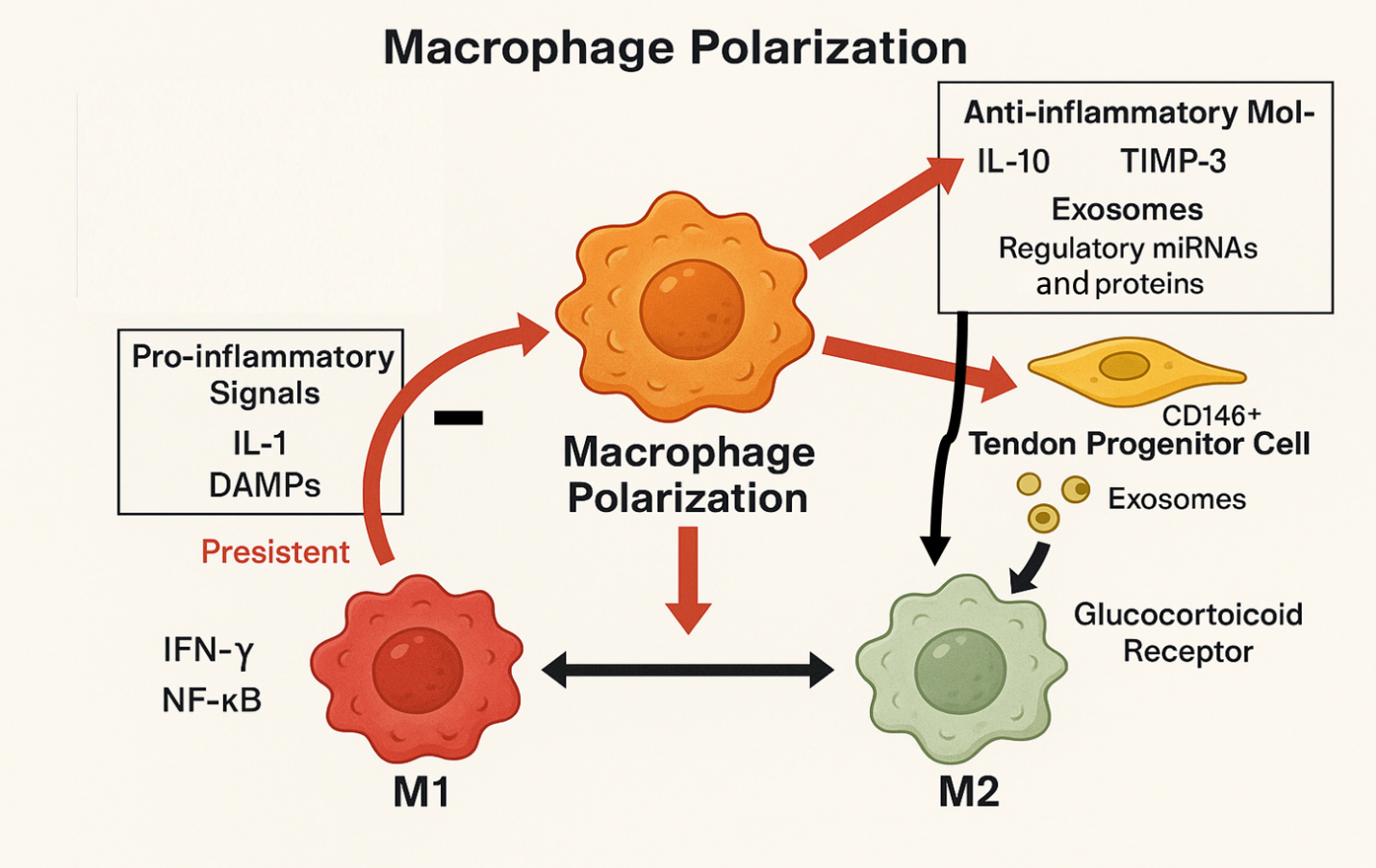
**

**Figure 3.** Schematic representation of macrophage polarization within the tendon microenvironment. Uncommitted macrophages can polarize into either M1 or M2 phenotypes in response to external cues. Persistent pro-inflammatory signals such as IL-1 and others drive macrophages toward the M1 state, maintaining an inflammatory environment. In contrast, tendon-derived CD146⁺ progenitor cells contribute to M2 polarization by secreting anti-inflammatory mediators (IL-10, TIMP-3) and releasing exosomes enriched with regulatory microRNAs and proteins. Inflammation-resolving pathways mediated by glucocorticoid receptor activation further support M2 polarization.


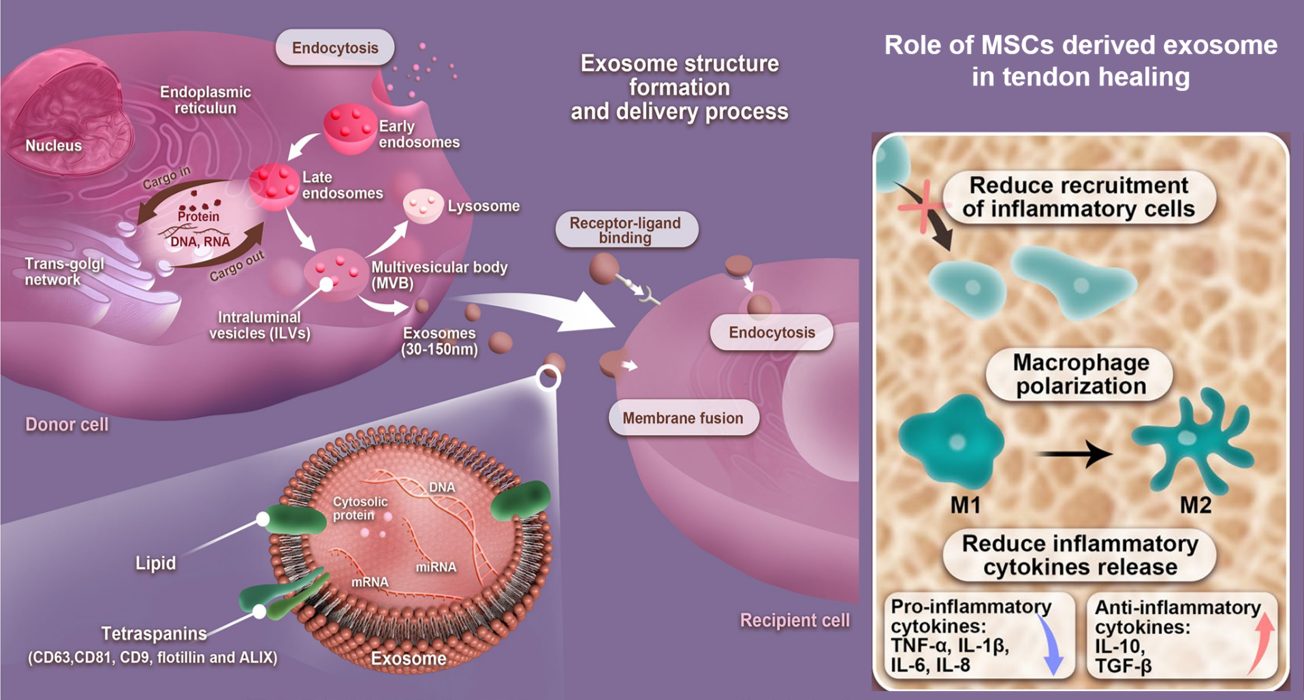


**Figure 4.** Illustration of exosome biogenesis, structure, and functional role in tendon healing. MSCs-derived exosomes carry regulatory proteins and RNAs, and upon delivery to recipient cells, they reduce inflammatory cell recruitment, shift macrophages from the M1 to M2 phenotype, and suppress pro-inflammatory cytokines (e.g., TNF-α, IL-1β) while enhancing anti-inflammatory mediators (e.g., IL-10, TGF-β) **(169)**.
